# A Novel Time Delay Estimation Algorithm for 5G Vehicle Positioning in Urban Canyon Environments

**DOI:** 10.3390/s20185190

**Published:** 2020-09-11

**Authors:** Zhongliang Deng, Xinyu Zheng, Hanhua Wang, Xiao Fu, Lu Yin, Wen Liu

**Affiliations:** School of Electronic Engineering, Beijing University of Posts and Telecommunications, Beijing 100876, China; dengzhl@bupt.edu.cn (Z.D.); whh0710@bupt.edu.cn (H.W.); xiaofu@bupt.edu.cn (X.F.); inlu_mail@bupt.edu.cn (L.Y.); liuwen@bupt.edu.cn (W.L.)

**Keywords:** vehicle positioning, 5G positioning, time delay estimation, multiple signal classification, super-resolution

## Abstract

Vehicle positioning with 5G can effectively compensate for the lack of vehicle positioning based on GNSS (Global Navigation Satellite System) in urban canyons. However, there is also a large ranging error in the non-line of sight (NLOS) propagation of 5G. Aiming to solve this problem, we consider a new time delay estimation algorithm called non-line of sight cancellation multiple signal classification (NC-MUSIC). This algorithm uses cross-correlation to identify and cancel the NLOS signal. Then, an unsupervised multipath estimation method is used to estimate the number of multipaths and extract the noise subspace. The MUSIC spectral function can be calculated by the noise subspace. Finally, the time delay of the direct path is estimated by searching the peak of MUSIC spectral function. This paper adopts the 5G channel model developed by 3GPP TR38.901 for simulation experiments. The experiment results demonstrated that the proposed algorithm has obvious advantages in terms of NLOS propagation for urban canyon environments. It provided a high-precision time delay estimation algorithm for observed time difference of arrival (OTDOA), joint angle of arrival (AOA) ranging, and other positioning methods in the 5G vehicle positioning method, which can effectively improve the positioning accuracy of 5G vehicle positioning in urban canyon environments.

## 1. Introduction

As a part of the vehicular network, vehicle positioning plays an important role in intelligent traffic management systems, vehicle detection, autonomous driving, intelligent parking, and so on [1]. Traditional vehicle positioning usually adopts global navigation satellite system (GNSS) represented by global positioning system (GPS), GLONASS, Beidou navigation system (BDS), and Galileo [2,3,4,5,6,7]. However, in urban canyon environments, GNSS signals are often blocked by high-rise buildings and perform with low capability in terms of positioning [8]. Mobile communication networks can fill the gaps in positioning performance of GNSS in urban environment due to its widely distribution and high signal transmission quality [9,10,11,12]. However, it lacks hear-ability (the capability of detecting the number of base stations). One of the striking features of fifth generation mobile networks (5G) is ultra-dense network (UDN) which improves the hear-ability of mobile communication network. Therefore, UDN brings the opportunity of 5G vehicle positioning in urban canyon environments. The 5G vehicle positioning method can be divided into the following categories: the method based on observed time difference of arrival (OTDOA) [13], angle of arrival (AOA)-based method [14,15], joint AOA ranging method [16,17], and so on. Both OTDOA and joint AOA-ranging methods require accurate time delay estimation of 5G signals to improve positioning performance.

The time delay estimation algorithm can be divided into two categories: correlation peak detection-based algorithm [12] and super-resolution-based algorithm [18,19,20,21,22]. Super-resolution-based algorithm has higher time delay estimation accuracy than correlation peak detection-based algorithm. As a popular algorithm of super-resolution-based algorithm, multiple signal classification (MUSIC) algorithm estimates time delay by the orthogonality between the steering vector of received baseband signal and noise subspace of received baseband signal’s covariance matrix. MUSIC based time delay estimation methods have been studied in recent years, Xinrong et al. [23] applied super-resolution spectral estimation techniques to the measured channel frequency response to accurately estimate TOA for indoor geolocation applications. The simulation results show that super-resolution techniques can significantly improve the performance of TOA estimation as compared with conventional techniques including direct IFT and DSSS signal-based cross-correlation techniques. Jing et al. [24] proposed a frequency-domain-based cross-spectral super-resolution time delay estimate algorithm, and the method performed an accurate time delay estimation in the multipath channel model that they proposed in the paper. Fang et al. [25] compared the performance of the super-resolution method with MUSIC and a traditional cross-correlation algorithm in a simulated indoor multipath TOA environment, the simulation results showed that TOA estimation with time domain channel estimation can approach high level accuracy and stability. Feng-Xiang et al. [26] converted time delay estimation into a sinusoidal parameter estimation problem, and the sinusoidal parameters are estimated by generalizing the MUSIC algorithm. Simulation results showed that the proposed algorithm performs better than the classical correlation approach and the conventional MUSIC method for the closely spaced components in multipath environments. Khanzada et al. [27] compared the estimation of signal parameters via the rotational invariance technique (ESPRIT), root-multiple signal classification (RootMUSIC), and matrix pencil (MP) for ranging. The simulation results show that RootMUSIC and MP algorithm demonstrate better performance in ranging. Wang et al. [28] used a two-dimensional MUSIC algorithm to estimate TOA and AOA in a IR-UWB system, and the simulation results showed that the performance is better than RootMUSIC and matrix pencil algorithm. Hailong et al. [29] proposed a novel TDOA algorithm with super resolution based on a multi-dimensional cross-correlation function, namely the volume cross-correlation function (VCC). Numerical simulations showed an excellent time resolution capability of the algorithm in multipath environment. Gao et al. [22] made comparisons of the super-resolution TOA/TDOA estimation algorithms and the experience showed that the MUSIC-CR algorithms have the highest resolution and lowest SNR threshold. Abudabbousa et al. [30] proposed a super-resolution TDOA estimate algorithm based on orthogonal frequency division multicarrier, the experimental results showed that the approach can be useful for several applications needing accurate positioning. Chen et al. [31] proposed two 2-D DOA and TOA estimators called FFT-MUSIC and two-step FFT-MUSIC. Simulation results showed that both estimators can reduce the computation cost by 1–2 orders of magnitude.

**Figure 1:**
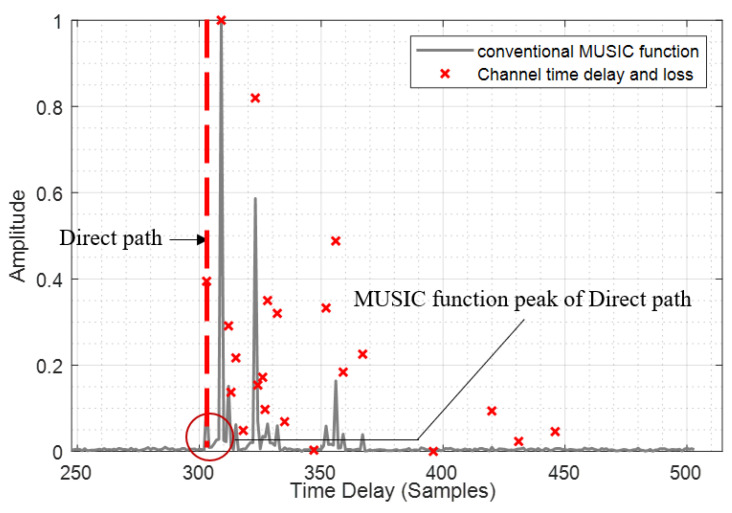
The conventional MUSIC spectral function in typical NLOS channel.

All the above methods effectively improve the accuracy and robustness of time delay estimation. However, few researchers have addressed the problem of non-line of sight (NLOS) propagation scenarios which is common for mobile communication signals in urban canyon environments. In some cases, the signal strength of direct path is weaker than non-direct path in NLOS propagation scenario, e.g., when there is an obstruction between the transmitting antenna and the receiving antenna, and the obstruction absorbs electromagnetic waves more heavily (such as thick walls), the direct path will receive severe degradation from the obstruction, while the NLOS path still has strong signal strength through the transmission to the receiving antenna. The direct path will be submerged by the non-direct path in conventional MUSIC algorithm and this problem will cause a large delay estimation error as shown in Figure 1. In Figure 1, we simulated a typical channel in an NLOS propagation scenario, and the peak of direct path in MUSIC spectral function of the received signal is submerged by the non-direct path. In order to solve this problem, we propose a novel time delay estimation algorithm called non-line of sight cancellation multiple signal classification (NC-MUSIC). In this algorithm, the time delay spread of received signal is roughly estimated through the cross-correlation method, and the NLOS path with strong signal strength is cancelled while the direct path is retained. This step will reduce the peak of NLOS path and make the peak of direct path more obvious. Then, the covariance matrix of the received signal is calculated and the eigenvalue decomposition (EVD) is carried out. An unsupervised multipath estimation method is used to estimate the number of multipath in the propagation channel. With the number of multipath and the EVD, the noise subspace of covariance matrix is solved. The MUSIC spectral function is calculated from the noise subspace. Finally, the time delay of the direct path is estimated by searching the peak of the MUSIC spectral function.

The contributions of this paper are:

- The proposal of a novel accurate time delay estimation algorithm based on NLOS cancellation and MUSIC super-resolution method.
- The proposal of an unsupervised multipath estimation method to estimate the multipath number of received baseband signal.
- Improvement of the MUSIC spectral function calculation method in conventional MUSIC super-resolution to make the peak of direct path more obvious.

The proposed algorithm in this paper is used to eliminate the NLOS error due to the signal propagation. In practice, there is still a synchronization error between the vehicle and the base station in addition to the NLOS error. This error will also lead to errors in the positioning results. The distance between RX and TX can be measured by round-trip time (RTT) and the synchronization error between RX and TX can also be ignored by calculating TDOA.

The rest of the paper is organized as follows. The system model is developed in Section 2. The NC-MUSIC algorithm is defined in Section 3. Then, the simulation results are discussed in Section 4. Finally, the conclusions and future work are drawn in Section 5.

## 2. System Model

In this section, the 5G baseband signal model and time delay estimate method based on conventional MUSIC will be presented.

### 2.1. 5G Baseband Signal Model

**Figure 2:**
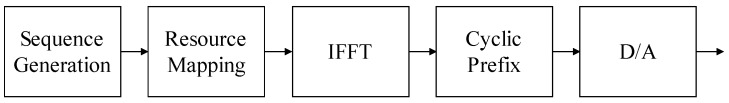
The procedure of 5G baseband signal generation.

According to 3GPP New Radio (NR) Technical Specification (TS) 38.211 [32], the downlink signal of 5G is generated based on cyclic prefix orthogonal frequency division multiplexing (CP-OFDM), and it is obtained by a stepwise procedure illustrated in Figure 2 and described below.

First, pseudo-random sequences are generated by a sequence generation module. Then, the pseudo-random sequence is mapped with the QPSK symbol to get a 5G reference signal sequence, and the reference signal sequence is mapped to resource elements in different ways according to different uses. Then, inverse fast Fourier transform (IFFT) is carried out on the resource elements to generate 5G time-domain signal, and the baseband signal is generated through cyclic prefix and digital-to-analog (D/A) conversion.

The pseudo-random sequence in TS38.211 adopts the 31-length gold code, which is generated as follows:

(1) $$\begin{array}{c} c(n)=(x_{1}(n+N_{C})+x_{2}(n+N_{C}))\mathrm{mod}2\\ x_{1}(n+31)=(x_{1}(n+3)+x_{1}(n))\mathrm{mod}2\\ x_{2}(n+31)=(x_{2}(n+3)+x_{2}(n+2)+x_{2}(n+1)+x_{2}(n))\mathrm{mod}2 \end{array}$$

where $N_{C}=1600$, the initialization of first gold sequence $x_{1}\left(n\right)$ is $x_{1}\left(1\right)=1$, $x_{1}\left(n\right)=0$, n=1,2,…,30, and the initialization of the second gold sequence $x_{2}\left(n\right)$ is $c_{init}=\sum_{i=0}^{30}x_{2}\left(i\right)\cdot 2^{i}$, $c_{init}$ depends on the purpose of the reference signal and mod2 is modulo-2 addition. The reference signal sequence r(m) is mapped by QPSK symbols from the 31-length gold sequence in the following way:

(2) $$r(m)=\frac{1}{\sqrt{2}}(1-2c(2m))+j\frac{1}{\sqrt{2}}(1-2c(2m+1)),$$

The generation method of 5G baseband signal in 3GPP TS38.211 is defined by

(3) $$\begin{array}{c} s_{l}^{\left(p,\mu \right)}\left(t\right)=\{\begin{array}{ll} {\bar{s}}_{l}^{\left(p,\mu \right)}\left(t\right) & t_{\mathrm{start},l}^{\mu }\leq t<t_{\mathrm{start},l}^{\mu }+T_{\mathrm{symb},l}^{\mu }\\ 0 & \mathrm{otherwise} \end{array}\\ {\bar{s}}_{l}^{\left(p,\mu \right)}\left(t\right)=\overset{N_{\mathrm{grid},x}^{\mathrm{size},\mu }N_{\mathrm{sc}}^{\mathrm{RB}}-1}{\underset{k=0}{\sum }}a_{k,l}^{\left(p,\mu \right)}e^{j2\pi \left(k+k_{0}^{\mu }-N_{\mathrm{grid},x}^{\mathrm{size},\mu }N_{\mathrm{sc}}^{\mathrm{RB}}/2\right)\Delta f\left(t-N_{\mathrm{CP},l}^{\mu }T_{c}-t_{\mathrm{start},l}^{\mu }\right)}\\ k_{0}^{\mu }=\left(N_{\mathrm{grid},x}^{\mathrm{start},\mu }+N_{\mathrm{grid},x}^{\mathrm{size},\mu }/2\right)N_{\mathrm{sc}}^{\mathrm{RB}}-\left(N_{\mathrm{grid},x}^{\mathrm{start},\mu_{0}}+N_{\mathrm{grid},x}^{\mathrm{size},\mu_{0}}/2\right)N_{\mathrm{sc}}^{\mathrm{RB}}2^{\mu_{0}-\mu }\\ T_{\mathrm{symb},l}^{\mu }=\left(N_{u}^{\mu }+N_{\mathrm{CP},l}^{\mu }\right)T_{c}, \end{array}$$

where $s_{l}^{\left(p,\mu \right)}\left(t\right)$ is the time-continuous signal on antenna port *p* and subcarrier spacing configuration μ for OFDM symbol $l\in \left\{0,1,\ldots ,N_{\mathrm{slot}}^{\mathrm{subframe},\mu }N_{\mathrm{symb}}^{\mathrm{slot}}-1\right\}$ in a subframe. $N_{\mathrm{grid},x}^{\mathrm{size},\mu }$ is the size of the resource grid, $N_{\mathrm{grid},x}^{\mathrm{start},\mu }$ is the start of the resource grid, $N_{\mathrm{sc}}^{\mathrm{RB}}$ is number of subcarriers per resource block, $a_{k,l}^{\left(p,\mu \right)}$ is the value of resource element (k,l) for antenna port p and subcarrier spacing configuration μ, $N_{\mathrm{CP},l}^{\mu }$ is cyclic prefix length, $T_{c}$ is basic time unit for NR, $\mu_{0}$ is the largest μ value among the subcarrier spacing configurations. Some of the configuration parameters are used for coordinate with other reference signals in 5G, and their values do not affect the waveform of the 5G positioning reference signal.

In order to facilitate the verification of the algorithm in this paper, we simplify the generation method of a 5G baseband signal, remove configuration parameters which are used for coordinate with other reference signals, and retain necessary parameters to generate 5G signal waveform. The simplified generation method of 5G signal baseband is as follows:

(4) $$s(t)=\sum_{m=0}^{N_{SC}-1}r(m)e^{j2\pi (m+m_{0})\Delta f(t-T_{CP})},$$

where s(t) is continuous analog baseband signal in time domain, $N_{SC}$ is the number of subcarriers, $m_{0}$ is the initial frequency offset, Δf is subcarrier space, and $T_{CP}$ is the time of cyclic prefix.

In practical application, 5G baseband signal is discrete digital signal generated by 5G baseband chip. In order to conform to the actual situation, 5G baseband signal is discretized:

(5) $$s_{D}(n)=\sum_{k=0}^{N_{SC}-1}r(k)e^{j2\pi (k+k_{0})\Delta f(nT_{C}-T_{CP})},$$

where $s_{D}\left(n\right)$ is discrete digital signal in time domain and $T_{C}$ is sample interval of baseband signal.

### 2.2. Time Delay Estimation Method

In a time delay estimation method, it is assumed that the discrete transmitted signal is $s_{T}\left(n\right)$ and the received signal transmitted through multipath channel is:

(6) $$s_{R}(n)=\sum_{k=0}^{N_{M}-1}\alpha_{k}s_{T}(n-\tau_{k})+\omega (n),$$

where $s_{R}\left(n\right)$ is received signal, $s_{T}\left(n\right)$ is transmit signal, $N_{M}$ is the number of paths in multipath channel, $\alpha_{k}$ and $T_{k}$ is the loss and time delay of the (k+1)th path in channel, ω(n) is noise. We express Equation (6) in matrix form:

(7) $$S_{R}=A\lambda +W,$$

where

(8) $$S_{R}={\left[\begin{array}{cccc} s_{R}(0) & s_{R}(1) & \cdots & s_{R}(N-1) \end{array}\right]}^{T},$$

(9) $$A=[\begin{array}{cccc} a(\tau_{0}) & a(\tau_{1}) & \cdots & a(\tau_{K-1}) \end{array}],$$

(10) $$a(\tau_{i})={\left[\begin{array}{cccc} s_{T}(0-\tau_{i}) & s_{T}(1-\tau_{i}) & \cdots & s_{T}(N-1-\tau_{i}) \end{array}\right]}^{T},$$

(11) $$\lambda =[\begin{array}{cccc} \alpha_{1} & \alpha_{2} & \cdots & \alpha_{K} \end{array}{]}^{T},$$

(12) $$W={\left[\begin{array}{cccc} \omega (0) & \omega (1) & \cdots & \omega (N-1) \end{array}\right]}^{T},$$

The covariance matrix of the received signal is

(13) $$R=E[S_{R}S_{R}^{H}],$$

The MUSIC super-resolution method is based on eigenvalue decomposition of the covariance matrix of the preceding received signal in (7)

(14) $$R=U_{S}\Lambda_{S}U_{S}^{H}+U_{e}\Lambda_{e}U_{e}^{H},$$

where $U_{S}$ is signal subspace, $U_{e}$ is noise subspace, and Λ is diagonal matrix whose elements are the eigenvalues of **R**. The following equation is given with the orthogonality between the steering vector of received baseband signal and noise subspace.

(15) $$a(\tau_{i})U_{e}^{H}=0,$$

Conventional MUSIC algorithm uses the following spectral function to estimate time delay.

(16) $$P_{MUSIC}=\frac{1}{a(\tau_{i})U_{e}^{H}U_{e}a^{H}(\tau_{i})},$$

The estimated time delay is:

(17) $$\hat{\tau }=\underset{\tau }{\arg}\left[\max\left(P_{MUSIC}\right)\right],$$

The MUSIC algorithm for delay estimation has superior resolution and can improve the ranging ability of the 5G OFDM signal.

## 3. Methodology

**Figure 3:**
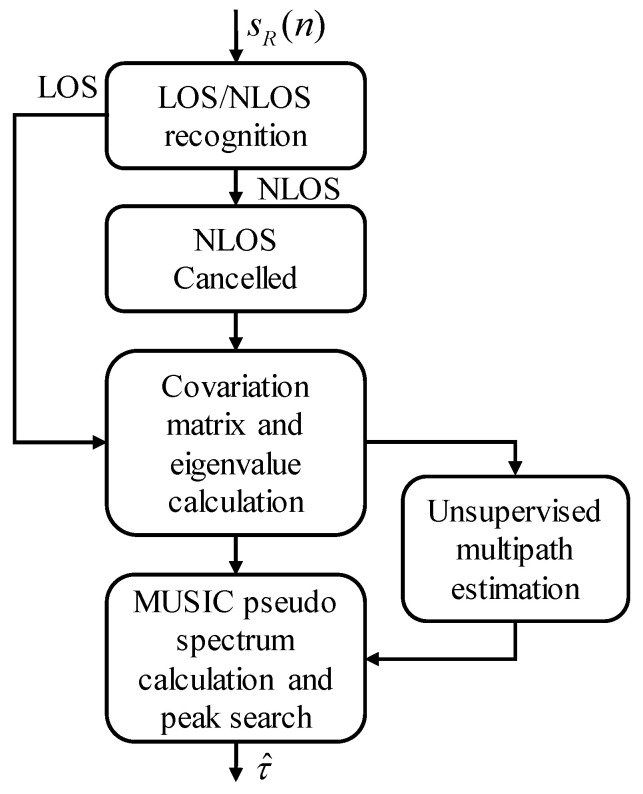
The structure diagram of the proposed NC-MUSIC algorithm.

This section introduces the proposed time delay estimation algorithm called non-line of sight cancellation multiple signal classification (NC-MUSIC). The structure diagram of the proposed NC-MUSIC algorithm is shown in Figure 3. First of all, NLOS/LOS is recognized by cross-correlation results of received signal, if the received signal is NLOS propagation, the non-direct paths will be cancelled according to the cross-correlation results. Then, MUSIC peak search is conducted on the NLOS cancelled signal with the multipath number estimated by unsupervised multipath estimation. And finally, the accurate estimation of direct signal propagation time delay is outputted. The following is a detailed description of the NC-MUSIC algorithm.

### 3.1. LOS/NLOS Identification and NLOS Cancellation

The received signal is cross-correlated, and the result of discrete cross-correlation under multi-path transmission is as follows:

(18) $$R_{A}(\tau )=\sum_{n=0}^{K_{A}-1}\left[s_{T}(n-\tau )s_{R}^{*}(n)\right]=\sum_{n=0}^{K_{A}-1}\left[s_{T}(n-\tau )\sum_{i}^{K}s_{T}^{*}(n-\tau_{i})\right],$$

(19) $$K_{A}=K_{R}+K_{T}-1,$$

where the $K_{R}$ is the length of discrete received baseband signal, $K_{T}$ is the length of discrete transmit baseband signal, and $K_{A}$ is the length of cross-correlation result of the transmit signal and received signal. Then, the peak of the discrete cross-correlation result was searched based on gradient to roughly estimate the multipath time delay. The gradient of cross-correlation result is:

(20) $$G_{A}(\tau )=R_{A}(\tau )-R_{A}(\tau -1),$$

The $G_{A}\left(\tau \right)$ is the gradient of cross-correlation result, the roughly time delay estimation result is:

(21) $$\tau_{i}=\underset{\tau }{\arg}\left[G_{A}\left(\tau -1\right)>0\wedge G_{A}\left(\tau \right)<0\wedge R_{A}\left(\tau \right)\geq P_{T}\right]\left(i=1,2,\ldots ,M_{p}\right),$$

where $\tau_{i}$ is the roughly time delay estimation result, $P_{T}$ is the threshold of the search of cross-correlation, the peak value less than this threshold is not regarded as multipath delay, and $M_{P}$ is the number of multipath time delay. The corresponding correlation peak value of each multipath delay is:

(22) $$P_{i}=R_{A}\left(\tau_{i}\right)\left(i=1,2,\ldots ,M_{P}\right),$$

After obtaining the multipath time delay estimation of received signal and the corresponding correlation peak, NLOS/LOS identification can be conducted. The identification principle is: if the correlation peak with the minimum time delay is not the maximum correlation peak, it means that the signal has been propagated by NLOS. For LOS component which is weaker than the NLOS components, the peak of the LOS path can be detected by correlation, but after using the conventional MUSIC algorithm, the peak of the LOS path is submerged by the peak of the NLOS path. This phenomenon can be seen in Figure 1, where the power of the LOS path is not very weak, but the LOS path in the spectral function of the conventional MUSIC algorithm is severely affected by the NLOS path, and the peaks are submerged and the LOS path cannot be identified, so the NLOS component needs to be cancelled. The way of NLOS cancel is to reconstruct the non-direct path signal. The method of reconstruction of non-direct path signal is as follows.

(23) $$s_{NLOS}=\sum_{i=1}^{M_{P}-1}Am_{i+1}s_{T}\left(n-\tau_{i+1}\right),$$

where $s_{NLOS}$ is the non-direct path signal, $Am_{i}$ is the amplitude loss of each non-direct path signal component, and the calculation method is as follows:

(24) $$Am_{i}=\frac{R_{A}\left(\tau_{i}\right)}{\max\left[\sum_{n=0}^{K_{A}-1}s_{T}\left(n-\tau \right)s_{T}^{*}\left(n\right)\right]},$$

Received signal minus the reconstruction of the non-direct path signal can get NLOS cancelled signal $s_{NC}\left(n\right)=s_{R}\left(n\right)-s_{NLOS}\left(n\right)$. There are residual non-direct path signals component because the cross-correlation method is not accurate for time delay estimation, and a second or multiple NLOS cancellations can be applied by Equations (20)–(24). However, after repeated cancellations, the correlation peak of weak signal delay will be confused with the side peak of OFDM signal correlation, which will lead to greater error. The experimental part will give a comparative analysis of cancellation times and ranging accuracy. The next subsection mainly describes the covariation matrix and eigenvalue calculation. In order to simplify the description of the algorithm, the signals cancelled by different cancellation times are denoted as $s_{NC}$.

### 3.2. Covariation Matrix and Eigenvalue Calculation

Since the signal after NLOS cancel still has direct path and multi-path residual components, it can be expressed as

(25) $$s_{NC}\left(n\right)=\sum_{i=0}^{N_{NC}-1}\beta_{i}s_{T}\left(n-\tau_{i}\right)+\omega \left(n\right)$$

where $N_{NC}$ is the number of multipath after NLOS cancel, $\beta_{i}$ is the amplitude loss of each multipath signal components, $\tau_{i}$ is the time delay of each multipath signal components. The baseband signal in Equation (5) is substituted into Equation (25) as the transmitting signal, and the following equation can be obtained:

(26) $$\begin{array}{c} s_{NC}\left(n\right)=\overset{N_{NC}-1}{\underset{i=0}{\sum }}\beta_{i}\left[\overset{N_{sc}}{\underset{k=0}{\sum }}r\left(k\right)\times e^{j2\pi \left(k+k_{0}\right)\Delta f\left[\left(n-\tau_{i}\right)T_{c}-T_{CP}\right]}\right]+\omega \left(n\right)\\ =\left[\begin{array}{cccc} \overset{N_{sc}-1}{\underset{k=0}{\sum }}r\left(k\right)e^{j2\pi \left(k+k_{0}\right)\Delta f\left[\left(-\tau_{0}\right)T_{c}-T_{CP}\right]} & \overset{N_{sc}-1}{\underset{k=0}{\sum }}r\left(k\right)e^{j2\pi \left(k+k_{0}\right)\Delta f\left[\left(-\tau_{1}\right)T_{c}-T_{CP}\right]} & \cdots & \overset{N_{sc}-1}{\underset{k=0}{\sum }}r\left(k\right)e^{j2\pi \left(k+k_{0}\right)\Delta f\left[\left(-\tau_{N_{NC}-1}\right)T_{c}-T_{CP}\right]}\\ \overset{N_{sc}-1}{\underset{k=0}{\sum }}r\left(k\right)e^{j2\pi \left(k+k_{0}\right)\Delta f\left[\left(1-\tau_{0}\right)T_{c}-T_{CP}\right]} & \overset{N_{sc}-1}{\underset{k=0}{\sum }}r\left(k\right)e^{j2\pi \left(k+k_{0}\right)\Delta f\left[\left(1-\tau_{1}\right)T_{c}-T_{CP}\right]} & \cdots & \overset{N_{sc}-1}{\underset{k=0}{\sum }}r\left(k\right)e^{j2\pi \left(k+k_{0}\right)\Delta f\left[\left(1-\tau_{N_{NC}-1}\right)T_{c}-T_{CP}\right]}\\ \vdots & \vdots & \ddots & \vdots \\ \overset{N_{sc}-1}{\underset{k=0}{\sum }}r\left(k\right)e^{j2\pi \left(k+k_{0}\right)\Delta f\left[\left(N-1-\tau_{0}\right)T_{c}-T_{CP}\right]} & \overset{N_{sc}-1}{\underset{k=0}{\sum }}r\left(k\right)e^{j2\pi \left(k+k_{0}\right)\Delta f\left[\left(N-1-\tau_{1}\right)T_{c}-T_{CP}\right]} & \cdots & \overset{N_{sc}-1}{\underset{k=0}{\sum }}r\left(k\right)e^{j2\pi \left(k+k_{0}\right)\Delta f\left[\left(N-1-\tau_{N_{NC}-1}\right)T_{c}-T_{CP}\right]} \end{array}\right]\\ \times \left[\begin{array}{c} \beta_{0}\\ \beta_{1}\\ \begin{array}{c} \vdots \\ \beta_{N_{NC}-1} \end{array} \end{array}\right]+\left[\begin{array}{c} \omega \left(1\right)\\ \begin{array}{c} \omega \left(2\right)\\ \vdots \end{array}\\ \omega \left(N-1\right) \end{array}\right] \end{array}$$

We can then write this signal model in vector form:

(27) $$S_{NC}=A\lambda +W$$

where:

(28) $$S_{NC}={\left[\begin{array}{cccc} s_{NC}(0) & s_{NC}(1) & \cdots & s_{NC}(N-1) \end{array}\right]}^{T}$$

(29) $$A=[\begin{array}{cccc} a(\tau_{0}) & a(\tau_{1}) & \cdots & a(\tau_{N_{NC}-1}) \end{array}]$$

(30) $$a\left(\tau_{i}\right)={\left[\begin{array}{cccc} \sum_{k=0}^{N_{sc}-1}r(k)e^{j2\pi (k+k_{0})\Delta f\left[\left(-\tau_{i}\right)T_{C}-T_{CP}\right]} & \sum_{k=0}^{N_{sc}-1}r(k)e^{j2\pi (k+k_{0})\Delta f\left[\left(1-\tau_{i}\right)T_{C}-T_{CP}\right]} & \cdots & \sum_{k=0}^{N_{sc}-1}r(k)e^{j2\pi (k+k_{0})\Delta f\left[\left(N-\tau_{i}-1\right)T_{C}-T_{CP}\right]} \end{array}\right]}^{T}$$

(31) $$\lambda =[\begin{array}{cccc} \beta_{0} & \beta_{1} & \cdots & \beta_{N_{NC}-1} \end{array}{]}^{T}$$

(32) $$W={\left[\begin{array}{cccc} \omega (0) & \omega (1) & \cdots & \omega (N-1) \end{array}\right]}^{T}$$

The covariance matrix R of the signal is calculated by $R=E\left[S_{NC}S_{NC}^{H}\right]$, and the eigenvalue decomposition of R is:

(33) $$R=U\Lambda U^{-1}$$

where, **U** is the matrix composed of the eigenvectors of R, and Λ is the diagonal matrix composed of the eigenvalues of R.

(34) $$\Lambda =\left[\begin{array}{cccc} \lambda_{1} & 0 & \cdots & 0\\ 0 & \lambda_{2} & \cdots & 0\\ \vdots & \vdots & \ddots & \vdots \\ 0 & 0 & \cdots & \lambda_{N} \end{array}\right]$$

Let $\lambda =\left[\lambda_{1}\;\lambda_{2}\ldots \;\lambda_{N}\right]$ be the set of eigenvalues of R. The next subsection describes the method of unsupervised multipath number estimation of received signal.

### 3.3. Unsupervised Multipath Estimation

The unsupervised multipath estimation part is used to estimate the number of multipath time delay to provide more accurate estimates. Eigenvalues could theoretically be gathered for two types of noise and signal eigenvalue, because the noise in the process of transmission is more stable, and it is so different between each signal path delay and signal loss. So, the noise subspace corresponding eigenvalues can be gathered for a cluster, the characteristics of the signal subspace corresponding values cannot be together for a cluster. The clustering results can be estimated in reverse. In other words, the outlier that cannot be clustered into a cluster can be taken as the eigenvalues corresponding to the signal subspace. The number of these outliers is the number of multipaths.

The number of clusters of noise eigenvalues is not a constant, so we must adopt a clustering method which doesn’t need the number of clusters. Moreover, all of the process is happening during the positioning, so a high complexity method shouldn’t be adopted. In this paper, the density-based spatial clustering of applications with noise (DBSCAN) [33] algorithm is adopted to cluster the eigenvalue λ because it doesn’t need to provide the number of clusters and has a low complexity. By reversely estimating the clustering results, the outliers that cannot be clustered as a class are regarded as the eigenvalues corresponding to the signal subspace, and the number of these outliers is the number of multipaths. Unsupervised multipath estimation is detailed below.

DBSCAN is a density-based clustering method. Its main parameters are clustering radius (*radius*) and the minimum number of elements in a class (*Pts*). Clustering radius affects the range of neighborhood density judgment. With the influence of clustering radius and the minimum number of elements in a class, the different values of the two parameters affect the threshold of the data density in the clustering. The data density above this threshold can be clustered into a class, while the data density below this threshold cannot be clustered into a class. In this paper, the data are the eigenvalues of the received signal covariance matrix, and the eigenvalues of the noise subspace have high similarity, in order to cluster the noise subspace eigenvalues with high similarity into one class and filter out the signal subspace eigenvalues with low similarity by clustering. We experimentally analyzed the values of *radius* and *Pts*, and found that signal subspace eigenvalues will be treated as noise subspace eigenvalues if the data density threshold is high, and noise subspace eigenvalues will be treated as signal subspace eigenvalues if the data density threshold is low, both of these circumstances will affect the accuracy of the final delay estimation results. So, we choose the appropriate radius and Pts to cluster the eigenvalues. In this paper, Pts=4 and *radius* is as follows:

(35) $$radius=\frac{\max\left(\lambda \right)-\min\left(\lambda \right)}{5\times 10^{4}}$$

The algorithm process of unsupervised multipath estimation is described as Algorithm 1. The estimated value of the output multipath number $N_{NC}$ is taken as the number of eigenvalues corresponding to the noise subspace in Equation (14), and the noise subspace $U_{e}$ is obtained.

**Algorithm1.** Unsupervised Multipath Estimation.**Input:λ***Pts*, *radius*
**Output:**
***N_NC_***
 1: Mark all elements of **λ** as unvisited; 2: Select an unvisited element *p* randomly; 3: Mark *p* as visited; 4: ***C_p_*** is a set whose elements’ distance from *p* is less than *radius*; 5: $N_{NC}\leftarrow length\left(C_{p}\right)$; 6: **IF**
$N_{p}\geq Pts$ 7:  Create a new cluster, add *p* to the cluster; 8:  **FOR** every element *p*′ in ***C_p_***; 9:   **IF**
*p*′ is unvisited 10:    Mark *p*′ as visited; 11:    ***C_p′_*** is a set whose elements’ distance from *p*′ is less than *radius*; 12:    **IF**
$length\left(C_{p\prime }\right)\geq Pts$ 13:     Add all elements of ***C_p′_*** to the new cluster; 14:    **END IF** 15:    **IF**
*p*′ doesn’t belong to any other cluster 16:     Add *p*′ to the new cluster; 17:    **END IF** 18:   **END IF** 19:  **END FOR** 20: **END IF** 21: ***N_mode_*** is the element number of the cluster with the largest number of elements; 22: $N_{NC}\leftarrow length\left(\lambda \right)-N_{mode}$

### 3.4. MUSIC Spectral Function Calculation and Peak Search

The noise subspace of the received signal is obtained through Equation (14). In order to reduce the submersion of the non-direct path signal to the direct path signal, we have improved the calculation method of the spectral function in the traditional MUSIC algorithm. The improved calculation method is as follows:

(36) $$P_{norm}=\left|a\left(\tau_{i}\right)U_{e}\right|$$

(37) $$P_{MUSIC}=10^{\left(\frac{\max(P_{norm})-P_{norm}}{\max(P_{norm})-\min(P_{norm})}\right)}$$

where $P_{MUSIC}$ is the spectral function.

Then, find the delay corresponding to the peak value of PMUSIC as the estimated value of propagation delay:

(38) $$\hat{\tau }=\arg\max(P_{MUSIC})$$

After the above process, the estimated propagation delay can be obtained.

## 4. Simulation Results

### 4.1. Simulation Scenario

This section discusses the ranging performance of proposed time delay estimation algorithm in NLOS channel model. In order to evaluate the performance of this method accurately, we adopted the 5G channel model developed by 3GPP TR38.901 [34] for simulation experiments. Two of the six types of UMi (urban micro) and UMa (urban macro) specified in the selection standard of experimental scenes, in which UMi scene means the base stations are mounted below rooftop levels of surrounding buildings and Uma scene means the base stations are mounted above rooftop levels of surrounding buildings. These two scenarios are used as the baseline for 5G positioning performance evaluation in 3GPP [35] and can fully represent the channels in urban canyon. The basic parameters for both scenarios are shown in Table 1. All simulations in this paper assume that TX and RX are rigorously time-synchronized so that errors due to time synchronization are not added when validating delay estimation errors. If the simulations do not assume that Tx and Rx are rigorously time-synchronized, then it is impossible to know whether the time delay error is caused by NLOS or by time synchronization. In practice, the errors due to time synchronization between the vehicle and the base station can be ignored by RTT or TDOA methods.

**Figure 4:**
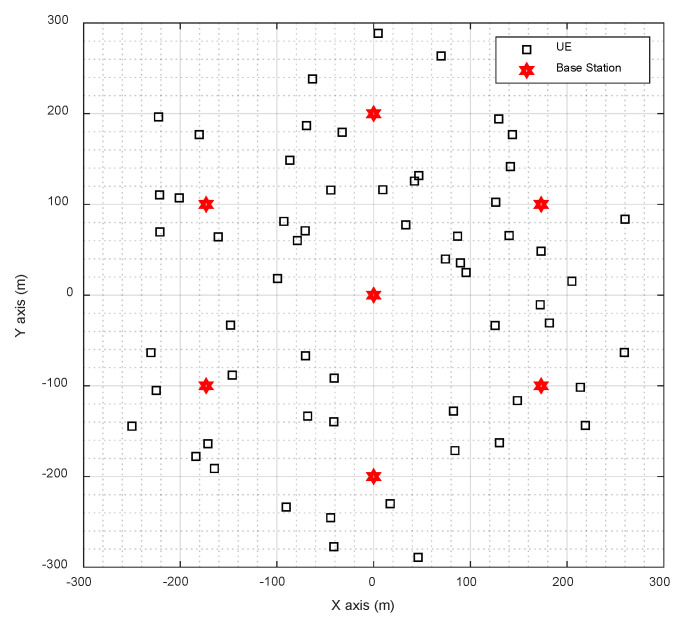
The distribution of base station and UE in UMi scenario.

**Figure 5:**
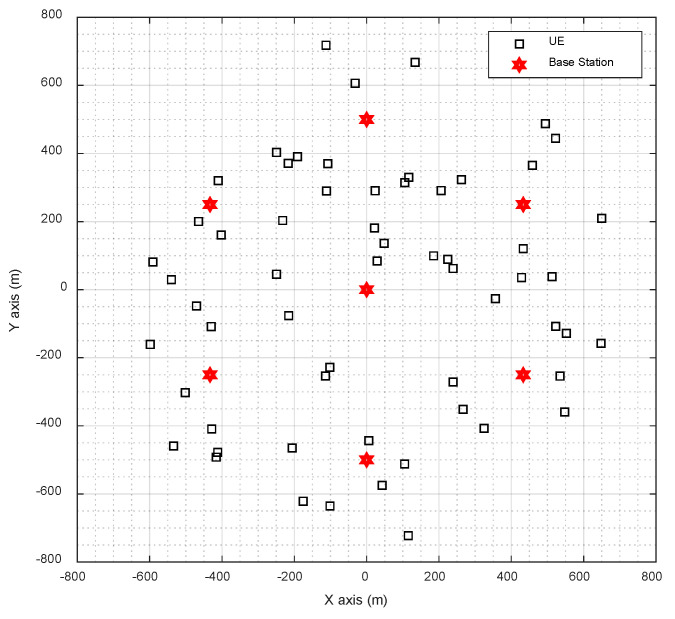
The distribution of base station and UE in UMa scenario.

In the simulation, the base station layout of cellular network is adopted. In both scenarios, seven base stations were arranged, and 60 UE were distributed randomly within the coverage area of seven base stations. The distribution of base station and UE is shown in Figure 4 and Figure 5.

**Figure 6:**
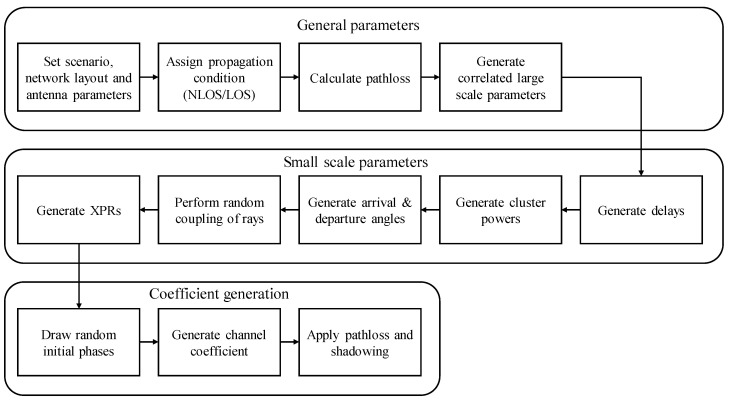
Channel model generation process in TR38.901.

Pathloss, LOS probability and penetration modelling are showed in detail in section 7.4 of 3GPP TR38.901. The channel generation process is shown in the Figure 6. The detailed channel generation process is given in section 7.5 of 3GPP TR38.901 [34].

In this paper the parameters of the simulation system are shown in Table 2, subcarrier spacing, transmission power, noise coefficient used the parameters specified in the TR38.855 Settings. The carrier frequency is 3.5 GHz due to the spectrum near the 3.5 GHz is commonly adopted by telecommunications operators, and 20 MHz, 50 MHz, 100 MHz are adopted as bandwidth. Moreover, 1, 2, and 3 times of cancellations are used for comparison. Finally, NC-MUSIC is compared with the conventional method.

### 4.2. Simulation Results

**Figure 7:**
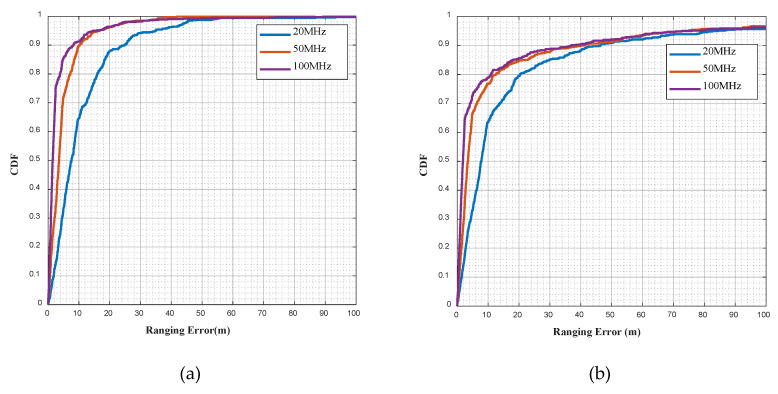
(**a**) CDF of the NC-MUSIC ranging error in UMi scenario with 20 MHz, 50 MHz and 100 MHz bandwidth; (**b**) CDF of the NC-MUSIC ranging error in UMa scenario with 20 MHz, 50 MHz and 100 MHz bandwidth.

Figure 7a,b show, respectively, the cumulative distribution functions (CDF) of the NC-MUSIC ranging error in UMi and UMa scenarios. In the simulation experiment, the signal bandwidth is 20 MHz, 50 MHz and 100 MHz, the time of cancellation is 1. It can be seen from the figures that the bandwidth is the main factor affecting the ranging error. The larger bandwidth is, the higher corresponding sampling rate, the higher time resolution, and the higher ranging accuracy are.

Table 3 and Table 4 are, respectively, the direct path identification rate of NC-MUSIC in UMi and UMa scenarios. The direct path identification rate means the probability that the result of time delay estimation is equal to the direct path delay. The direct path identification criteria stipulates that if the time difference between the estimated time delay and the first arrival time delay of the receiver signal in the channel does not exceed a sampling time, it is considered that the estimated delay accurately identifies the direct path. It can be seen from the tables that bandwidth and direct path identification rate do not show a simple positive correlation or negative correlation, and analysis shows that the greater bandwidth results in a higher time resolution. When there is a NLOS path with strong power and the time delay is similar to the direct path, the correlation calculation before NLOS cancellation cannot distinguish this NLOS path, so it is impossible to cancel this NLOS path. When the time delay is estimated with super resolution, this NLOS path will severely affect the direct path and submerge the peak of direct path, because of the phenomenon in Figure 1, thus this NLOS path will be mistaken as the direct path, leading to the decrease of the direct path identification rate.

**Figure 8:**
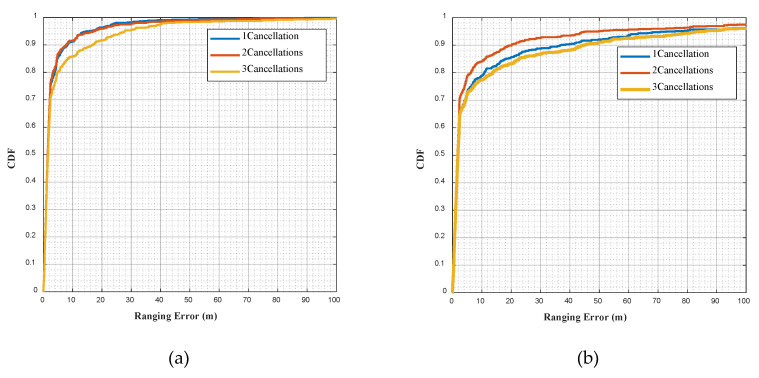
(**a**)CDF of the NC-MUSIC ranging error in UMi scenario with the cancellation time in the set {1,2,3}. (**b**) CDF of the NC-MUSIC ranging error in UMa scenario with the cancellation time in the set {1,2,3}.

Figure 8a,b show, respectively, the cumulative distribution functions (CDF) of the NC-MUSIC ranging error in UMi and UMa scenarios. Table 5 and Table 6 show, respectively, the direct path identification rate of NC-MUSIC in UMi and UMa scenarios. In the simulation experiment, the signal bandwidth is 100 MHz, while the direct path identification rate of one time cancellation and two time cancellation is similar. However, after three times of cancellation, the range error significantly increases and the identification rate of direct path decreases, the reason is analyzed as follows: in the third cancellation, the first two times of the direct path of NLOS signal were mistakenly cancelled, resulting in a large delay estimation error. In the UMa scenario, the direct path identification rate of two times of cancellation is higher than the direct path identification rate of one time of cancellation and three times cancellation. The main reason for this is that there are two NLOS paths with similar time delay. In correlation, only one of the two NLOS paths was identified and cancelled due to insufficient resolution. During the second NLOS cancellation, this NLOS path was identified and cancelled.

**Figure 9:**
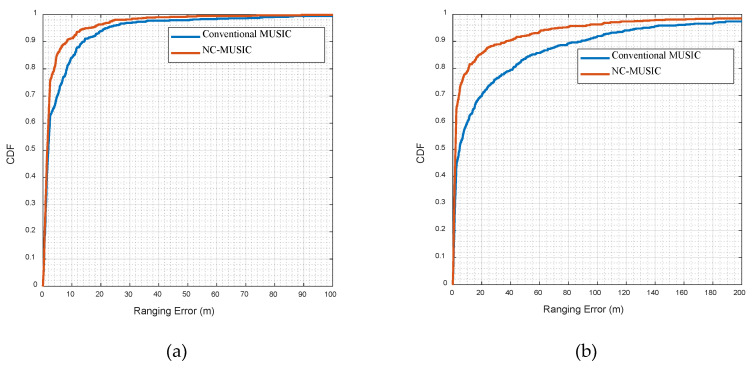
(**a**) CDF of the NC-MUSIC and conventional MUSIC ranging error in UMi scenario. (**b**)CDF of the NC-MUSIC and conventional MUSIC ranging error in UMa scenario.

Figure 9a,b show, respectively, a comparison of cumulative distribution functions (CDF) between the NC-MUSIC and conventional MUSIC ranging error in UMi and UMa scenarios. Table 7 and Table 8 show, respectively, the comparison of direct path identification rate between NC-MUSIC and conventional MUSIC in UMi and UMa scenarios. In the simulation experiment, the signal bandwidth is 100 MHz and the time of cancellation is 1. It can be seen from the figures and tables that the algorithm proposed in this paper is superior to the conventional MUSIC algorithm in ranging error and direct path identification rate both in UMi and Uma scenarios, and has good anti-NLOS ability and ranging performance in urban environment.

### 4.3. The Spectral Function of NC-MUSIC Algorithm in the Simulation

**Figure 10:**
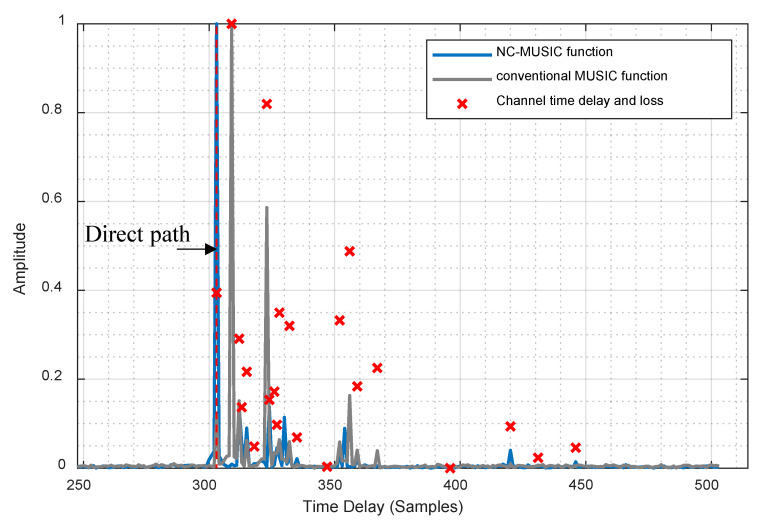
The spectral function of NC-MUSIC algorithm in TR38.901 channel model.

In order to intuitively show the practicability of the NC-MUSIC algorithm in this paper, we put the spectral function of NC-MUSIC algorithm. The spectral function of NC-MUSIC is compared with conventional MUSIC spectral function. The spectral function of NC-MUSIC in TR38.901 channel model is shown in Figure 10, and the simulation parameters in Table 9.

As can be seen from the figures, when time delay estimation is carried out under NLOS channel, the conventional MUSIC algorithm has taken the non-direct path with the largest strength as time delay, while the direct path with lower strength is greatly submerged in the conventional MUSIC calculation method, which was difficult to be identified in the spectral function peak search process, so the conventional MUSIC algorithm has a large delay estimation error under NLOS channel. The NC-MUSIC algorithm proposed in this paper canceled the three non-direct paths of path2, path3, path4, so as to accurately captured the direct path with lower strength, and thus it has a better performance for time delay estimation in the NLOS channel.

### 4.4. Analysis of the Impact of Estimation Error of Delay and Amplitude of NLOS Signal

**Figure 11:**
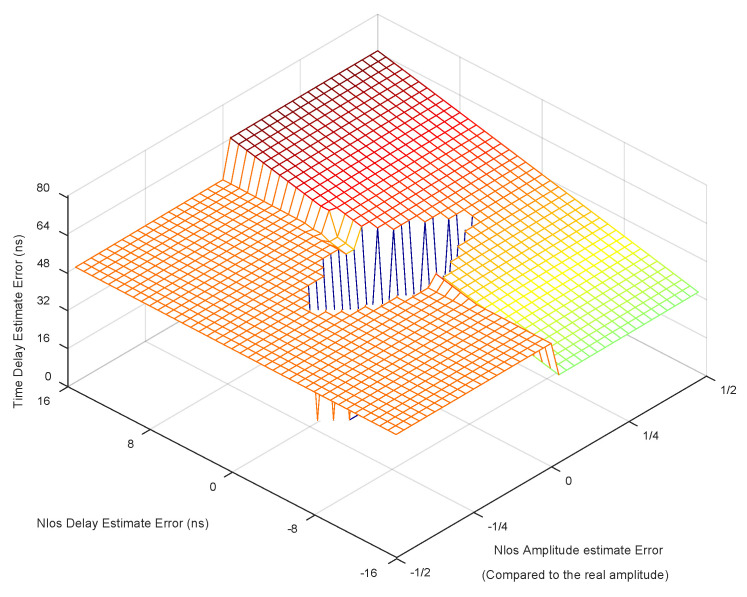
The impact of estimation error of delay and amplitude of NLOS signal.

In the NC-MUSIC algorithm proposed in this paper, the accuracy of NLOS cancellation impacts the final delay estimation accuracy, so we analyzed the impact of NLOS delay estimation accuracy and NLOS amplitude estimation accuracy on the final delay estimation accuracy, and we use the channel model in Section 4.3 for simulation experiment. The simulation results are shown in Figure 11, where the unit of error in NLOS signal delay estimation is ns and the unit of error in NLOS signal amplitude estimation is the true NLOS signal amplitude in the channel model.

As can be seen from the figure, when the delay estimation error and amplitude estimation error of NLOS signal are within a certain range, the algorithm can still effectively provide high-precision delay estimation, but when the delay estimation error and amplitude estimation error of NLOS signal are large, the signal will not only fail to eliminate the non-line-of-sight error, but also introduce multipath when subtracting the erroneous NLOS signal, making the delay estimation accuracy decrease.

## 5. Conclusions

In this paper, in order to solve the problem of large delay estimation error caused by NLOS in 5G vehicle positioning for modern urban canyon environments, a time-delay estimation algorithm based on MUSIC for NLOS propagation is proposed, namely NC-MUSIC. The algorithm improves the MUSIC algorithm under the non-line-of-sight transmission spectral function peak submergence problem. First of all, the received signal is calculated, before NLOS transmission is identified, and the NLOS paths reconstructed to eliminate the NLOS paths of received signal through the unsupervised multipath estimation method to calculate the noise subspace of the received signal. Through the calculation of the noise subspace spectral function, we improve the calculation method of spectral function in MUSIC algorithm is used to emphasize the non-line-of-sight under direct transmission signal peak. Through simulation experiments, it can be seen that the NC-MUSIC algorithm proposed in this paper has obvious advantages in terms of NLOS propagation for a modern urban canyon environment. It provides a high-precision time delay estimation algorithm for OTDOA, TOA+AOA, and other positioning methods in the 5G vehicle positioning method, which can effectively improve the positioning accuracy of 5G vehicle positioning in the modern urban environment.

## Figures and Tables

**Table 1:** Basic parameters in UMi and UMa.

Scenario	Tx Height	Rx Height	ISD (Intersite Distance)
Umi	10 m	1.5–2.5 m	200 m
Uma	25 m	1.5–2.5 m	500 m

**Table 2:** The parameters of the simulation system.

Parameters	Values
Scenario	UMi, UMa
Carrier frequency	3.5 GHz
Bandwidth	20 MHz, 50 MHz, 100 MHz
Subcarrier spacing (kHz)	15 kHz for 20 MHz and 50 MHz, 30 kHz for 100 MHz
Transmit power	23 dBm
Base station noise figure	5 dB
UE noise figure	9 dB
Pts	4

**Table 3:** Direct path identification rate of NC-MUSIC in UMi scenario with 20 MHz, 50 MHz and 100 MHz bandwidth.

Bandwidth	Direct Path Identification Rate	False Positive Rate	False Negative Rate
20 MHz	87.22%	0.69%	12.08%
50 MHz	89.44%	0.00%	10.56%
100 MHz	85.00%	0.14%	14.86%

**Table 4:** Direct path identification rate of NC-MUSIC in UMa scenario with 20 MHz, 50 MHz and 100 MHz bandwidth.

Bandwidth	Direct Path Identification Rate	False Positive Rate	False Negative Rate
20 MHz	78.89%	0.83%	20.28%
50 MHz	76.81%	0.00%	23.19%
100 MHz	72.22%	0.56%	27.22%

**Table 5:** Direct path identification rate of NC-MUSIC in UMi scenario.

Cancellations	Direct Path Identification Rate	False Positive Rate	False Negative Rate
1	85.00%	0.14%	14.86%
2	85.83%	0.69%	13.47%
3	80.00%	0.42%	19.58%

**Table 6:** Direct path identification rate of NC-MUSIC in UMa scenario.

Cancellations	Direct Path Identification Rate	False Positive Rate	False Negative Rate
1	72.22%	0.56%	27.22%
2	77.92%	0.28%	21.81%
3	71.39%	0.42%	28.19%

**Table 7:** Direct path identification rate of NC-MUSIC and conventional MUSIC in UMi scenario.

Methods	Direct Path Identification Rate
Conventional MUSIC	69.86%
NC-MUSIC	85.00%

**Table 8:** Direct path identification rate of NC-MUSIC and conventional MUSIC in UMa scenario.

Methods	Direct Path Identification Rate
Conventional MUSIC	51.11%
NC-MUSIC	72.22%

**Table 9:** The parameters of the simulation system.

Parameters	Values
Scenario	UMa
Carrier frequency	3.5 GHz
Bandwidth	100 MHz
Subcarrier spacing (kHz)	30 kHz
Transmit power	23 dBm
Base station noise figure	5 dB
UE noise figure	9 dB
Pts	4
